# Supplementary material for: The Effect of Minimum Unit Pricing for Alcohol on Prescriptions for Treatment of Alcohol Dependence: A Controlled Interrupted Time Series Analysis
Source: Int J Ment Health Addict. 2023 May 22:1–16. Online ahead of print. doi: 10.1007/s11469-023-01070-6 (PMC10202053; doi:10.1007/s11469-023-01070-6)
Supplement: Supplementary file 1 — Supplementary Material 1 [file 11469_2023_1070_MOESM1_ESM.docx]

**Supplementary material**

## Smoothing procedure

a) Computation of the average daily number of prescriptions by month

b) Difference between number of prescriptions recorded the last day of the month and a)

c) b) divided by the number of days with observations in every month

d) adding c) to the initial number of prescriptions per day.

–except for the last day of the month which is the mean of prescriptions during the month plus c)-

## Overall monthly volume of acamprosate, disulfiram, nalmefene and naltrexone in Scotland


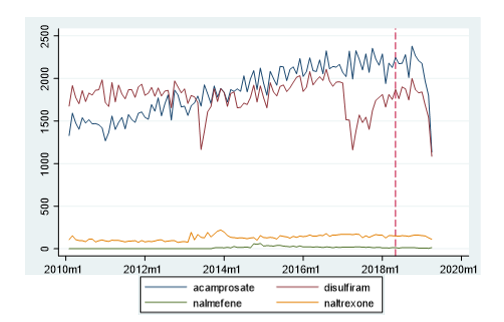


## Test common trend assumption

**Tot prescriptions**

------------------------------------------------------------------------------

diffTOT | Coefficient std. err. z P>|z| [95% conf. interval]

-------------+----------------------------------------------------------------

time | -.0002399 .0002949 -0.81 0.416 -.0008179 .000338

_cons | 1.905351 .0437738 43.53 0.000 1.819556 1.991146

**Prescription in most socioeconomically deprived decile**

------------------------------------------------------------------------------

diffsimd1 | Coefficient std. err. z P>|z| [95% conf. interval]

-------------+----------------------------------------------------------------

time | -.0012196 .0002039 -5.98 0.000 -.0016192 -.0008199

_cons | 2.340103 .0401737 58.25 0.000 2.261364 2.418842

-------------+----------------------------------------------------------------

**Prescription in least socioeconomically deprived deciles**

-------------+----------------------------------------------------------------

diffsimd210 | Coefficient std. err. z P>|z| [95% conf. interval]

-------------+----------------------------------------------------------------

time | .0003655 .000252 -1.45 0.147 -.0008594 .0001283

_cons | 1.675193 .0520892 32.16 0.000 1.578471 1.777286

-------------+----------------------------------------------------------------

**Tot New Patients**

------------------------------------------------------------------------------

diffpattot | Coefficient std. err. z P>|z| [95% conf. interval]

-------------+----------------------------------------------------------------

time | -.065017 .0148052 -4.39 0.000 -.0940346 -.0359994

_cons | 31.97473 2.004788 15.95 0.000 28.04541 35.90404

-------------+----------------------------------------------------------------

**Tot new patients in most socioeconomically deprived decile**

------------------------------------------------------------------------------

diffpatsimd1 | Coefficient std. err. z P>|z| [95% conf. interval]

-------------+----------------------------------------------------------------

time | -.0085571 .0065061 -1.32 0.188 -.0213089 .0041947

_cons | 5.288891 .7887012 6.71 0.000 3.743065 6.834717

-------------+----------------------------------------------------------------

**Tot new patients in least socioeconomically deprived decile**

--------------------------------------------------------------------------------

diffpatsimd210 | Coefficient std. err. z P>|z| [95% conf. interval]

---------------+----------------------------------------------------------------

time | -.0536921 .0131914 -4.07 0.000 -.0795467 -.0278374

_cons | 27.26895 1.818133 15.00 0.000 23.70547 30.83243

---------------+----------------------------------------------------------------

## Results on the difference of the series with common trend

**Tot prescriptions (difference of the log)**

diffTOT | Coefficient std. err. z P>|z| [95% conf. interval]

-------------+----------------------------------------------------------------

time | -.0003067 .0001911 -1.61 0.108 -.0006812 .0000678

MUP | .0097811 .0332375 0.29 0.769 -.0553632 .0749254

time_MUP | -.0009175 .0006695 -1.37 0.171 -.0022297 .0003946

**Prescriptions in least socioeconomically deprived deciles (difference of the log)**

diffsimd2_10 | Coefficient std. err. z P>|z| [95% conf. interval]

-------------+----------------------------------------------------------------

time | .0001363 .0002311 0.23 0.818 -.0003187 .0004037

MUP | .0147778 .0291533 0.44 0.659 -.0509352 .0804908

time_MUP | -.0019768 .0006312 -2.69 0.007 -.0034178 -.0005359

**New patients in the least socioeconomically deprived deciles (difference in actual values)**

--------------------------------------------------------------------------------

diffpatsimd210 | Coefficient std. err. z P>|z| [95% conf. interval]

---------------+----------------------------------------------------------------

time | -.0633426 .0136326 -4.65 0.000 -.090062 -.0366233

MUP | 4.57245 3.227303 1.42 0.157 -1.752948 10.89785

time_MUP | .0499487 .0413048 1.21 0.227 -.0310071 .1309045

*Falsification test for the difference whenever a MUP term was significant*

Intervention Pre-6month- in least socioeconomically deprived deciles

diffsimd210 | Coefficient std. err. z P>|z| [95% conf. interval]

-------------+----------------------------------------------------------------

time | -.0001737 .0002925 -0.59 0.553 -.0007469 .0003996

MUP | .0739486 .0439116 1.68 0.092 -.0121166 .1600139

time_MUP | -.0016832 .0006668 -2.52 0.012 -.0029901 .0003763

Intervention Post-6month- in least socioeconomically deprived deciles

diffsimd210 | Coefficient std. err. z P>|z| [95% conf. interval]

-------------+----------------------------------------------------------------

time | .0001766 .0002194 0.81 0.421 -.0002533 .0006065

MUP | .0235616 .0373441 0.44 0.528 -.0967547 .0496315

time_MUP | -.0016172 .0008612 -1.88 0.060 -.0033052 .0000707

## Change in models with additional variables for shortage in disulfiram whenever main analysis had a MUP significant term

## New patients in overall population

### **Shortage in disulfiram and time from shortage in disulfiram with MUP terms**

------------------------------------------------------------------------------

lalcpat | Coefficient std. err. z P>|z| [95% conf. interval]

-------------+----------------------------------------------------------------

lalcpat |

time | -.0019058 .0002339 -8.15 0.000 -.0023642 -.0014474

shortage | .0320825 .0345792 0.93 0.354 -.0356915 .0998565

time shortage| -.0008758 .0023383 -0.37 0.708 -.0054587 .0037071

MUP | .0700968 .0545766 1.28 0.199 -.0368713 .1770649

time_MUP | .0026715 .0022418 1.19 0.233 -.0017225 .0070654

_cons | 4.227051 .0225547 187.41 0.000 4.182844 4.271257

-------------+----------------------------------------------------------------

### **Shortage in disulfiram and time from shortage in disulfiram without MUP terms**

------------------------------------------------------------------------------

lalcpat | Coefficient std. err. z P>|z| [95% conf. interval]

-------------+----------------------------------------------------------------

lalcpat |

time | -.002022 .0001755 -11.52 0.000 -.002366 -.001678

shortage| .0447668 .0293158 1.53 0.127 -.0126911 .1022247

time shortage| .0020514 .0004768 4.30 0.000 .0011169 .0029859

_cons | 4.234248 .0203705 207.86 0.000 4.194323 4.274174

## New patients in least deprived groups

### **Shortage in disulfiram and time from shortage in disulfiram with MUP terms**

------------------------------------------------------------------------------

patalc2_10 | IRR std. err. z P>|z| [95% conf. interval]

-------------+----------------------------------------------------------------

time | .9982395 .000269 -6.54 0.000 .9977125 .9987668

shortage | 1.012224 .0423313 0.29 0.771 .9325652 1.098687

time shortage | .9984299 .0028533 -0.55 0.582 .9928532 1.004038

MUP | 1.054346 .0710281 0.79 0.432 .9239329 1.203168

time_MUP | 1.00358 .0027832 1.29 0.198 .99814 1.00905

_cons | 53.66015 1.425201 149.95 0.000 50.93827 56.52748

### **Shortage in disulfiram and time from shortage in disulfiram without MUP terms**

------------------------------------------------------------------------------

patalc2_10 | IRR std. err. z P>|z| [95% conf. interval]

-------------+----------------------------------------------------------------

time | .9980405 .0002146 -9.12 0.000 .99762 .9984612

shortage | 1.036207 .0381752 0.97 0.334 .9640229 1.113797

time shortage | 1.001993 .0005803 3.44 0.001 1.000856 1.003131

_cons | 54.31425 1.344907 161.33 0.000 51.74122 57.01523

------------------------------------------------------------------------------
